# Response to Initial Therapy of Differentiated Thyroid Cancer Predicts the Long-Term Outcome Better than Classical Risk Stratification Systems

**DOI:** 10.1155/2014/591285

**Published:** 2014-07-08

**Authors:** Albert Cano-Palomares, Ignasi Castells, Ismael Capel, Maria Rosa Bella, Santi Barcons, Angel Serrano, Xavier Guirao, Mercedes Rigla

**Affiliations:** ^1^Endocrinology and Nutrition Department, Parc Taulí Sabadell University Hospital, Parc Taulí s/n, Sabadell, 08208 Barcelona, Spain; ^2^Endocrinology, Diabetes and Nutrition Unit, Granollers General Hospital, Francesc Ribas s/n, Granollers, 08402 Barcelona, Spain; ^3^Universitat Internacional de Catalunya (UIC), Josep Trueta s/n, Sant Cugat, 08195 Barcelona, Spain; ^4^Pathology Department, Parc Taulí Sabadell University Hospital, Sabadell, Barcelona, Spain; ^5^Surgery Department, Parc Taulí Sabadell University Hospital, Sabadell, Barcelona, Spain; ^6^Pathology Department, Granollers General Hospital, Granollers, Barcelona, Spain; ^7^Surgery Department, Granollers General Hospital, Granollers, Barcelona, Spain

## Abstract

*Objective*. Although differentiated thyroid cancer (DTC) usually has an indolent course, some cases show a poor prognosis; therefore, risk stratification is required. The objective of this study is to compare the predictive ability of classical risk stratification systems proposed by the European Thyroid Association (ETA) and American Thyroid Association (ATA) with the system proposed by Tuttle et al. in 2010, based on the response to initial therapy (RIT). *Methods*. We retrospectively reviewed 176 cases of DTC with a median follow-up period of 7.0 years. Each patient was stratified using ETA, ATA, and RIT systems. Negative predictive value (NPV) and positive predictive value (PPV) were determined. The area under receiver operating characteristic (ROC) curve was calculated in order to compare the predictive ability. *Results*. RIT showed a NPV of 97.7%, better than NPV of ETA and ATA systems (93.9% and 94.9%, resp.). ETA and ATA systems showed poor PPV (40.3% and 41%, resp.), while RIT showed a PPV of 70.8%. The area under ROC curve was 0.7535 for ETA, 0.7876 for ATA, and 0.9112 for RIT, showing statistical significant differences (*P* < 0.05). *Conclusions*. RIT predicts the long-term outcome of DTC better than ETA/ATA systems, becoming a useful system to adapt management strategies.

## 1. Introduction

The incidence of differentiated thyroid cancer (DTC) has been increasing during the last few decades, mostly attributable to better detection of small papillary cancer as a result of improved diagnostic accuracy [1]. However, improved detection does not fully explain significant increase in tumours with adverse pathologic characteristics [2].

Despite the fact that most of DTC have an indolent course with conventional therapy (surgical treatment, ablative I131, and suppressive treatment with L-thyroxine), metastatic, recurrent, and poorly differentiated tumours still represent a challenge for the clinicians. In order to improve management disease, different groups have been focused on molecular pathways involved in thyroid cancer pathogenesis, thus providing prognostic markers for well-differentiated tumours, as well as new targets for therapy [3].

In DTC, the risk of recurrence/persistence is higher than the risk of disease-specific mortality. Different staging systems (American Joint Cancer Committee/Union Internationale Contre le Cancer) have been proposed to predict the risk of death. However, these systems do not predict accurately the risk of recurrence or persistent disease. The European Thyroid Association (ETA) in 2006 and the American Thyroid Association (ATA) in 2009 published management guidelines based on individualised risk stratification [4, 5]. Both of them incorporate data from tumour-related factors, clinical features, results of first posttherapy radioiodine whole-body scan, serum thyroglobulin (TG) measurement to assess the risk of recurrence and mortality, and also the need for adjuvant therapies and the early follow-up strategies. Moreover, during the last few years, several authors have proposed incorporating TG determination at the time of radioiodine ablation as predictive factor for the risk of recurrence or persistence disease in the postoperative period [6, 7].

Recently, different groups have been focused on new ongoing risk stratification systems incorporating variables that assess the response to initial therapy (RIT). These variables modify the initial risk estimation and, therefore, are able to achieve more accurate predictions of clinical outcomes. In 2010, Tuttle et al. developed a system that included data from the first two years of follow-up, period of time in which much of the variability in outcome can be appreciated, categorizing the response to therapy into excellent, acceptable, or incomplete [8]. An excellent response was defined as stimulated and suppressed TG <1 ng/mL and no evidence of disease by imaging. An acceptable response was defined as suppressed TG <1 ng/mL, the presence of low serum stimulated TG level (1–10 ng/mL), or the presence of nonspecific changes in neck ultrasound or nuclear medicine imaging. Finally, an incomplete response was defined as suppressed TG ≥1 ng/mL, stimulated TG ≥10 ng/mL, rising TG values, or persistent/newly identified disease on imaging.

The aim of our study is to compare the predictive value of the classical risk stratifications systems and the new system proposed by Tuttle et al. in our population.

## 2. Material and Methods

We retrospectively reviewed 176 patients with DTC evaluated at Granollers General Hospital and Parc Taulí Sabadell University Hospital between 2000 and 2012. The median follow-up period was 7.0 years (range 0.9–22.7).

All patients underwent total or near-total thyroidectomy. Compartment-oriented microdissection of lymph nodules was performed in case of preoperative suspected or intraoperative proven lymph nodules metastases. They also underwent thyroid ablation with ^131^I and received thyrotropin-suppressive treatment with L-thyroxine (LT4). Our cohort did not include patients classified as very low risk by the European system, because management guidelines do not recommend total or near-total thyroidectomy and thyroid ablation with ^131^I, making follow-up strategies impossible by TG assay and ^131^I whole body scan. We also excluded patients with inadequate follow-up information and positive anti-TG antibodies (TgAb) interfering in the TG assessment by immunometric methods.

Patients were followed up every 6–12 months and management and follow-up protocol was based on the European consensus published in 2006. We only included patients who had information available about results of neck ultrasound (US), performed, suppressed, and stimulated TG determinations, and at least one diagnostic ^131^I whole body scan, during the first two years of follow-up.

Before 2002, different TG assays were used with a functional sensitivity of approximately 1 ng/mL. Starting in 2002, all TG values were tested by solid-phase immunochemiluminometric assay with an analytical sensitivity of 0.2 ng/mL and a functional sensitivity of 0.9 ng/mL normalized to Certified Reference Material 457 (Immulite, Siemens, Inc.). Both stimulated and suppressed TG were tested by the same assay. The TgAb levels were measured by immunometric assay with the lowest reportable concentration of 10 IU/mL.

After initial treatment, each patient was stratified using the American Joint Cancer Committee/Union Internationale Contre le Cancer (AJCC/UICC) staging system and the ATA and the ETA systems. All data obtained after the first whole body scan and stimulated TG was used to stratify patients into an excellent, acceptable, or incomplete response to initial therapy.

At the end of the follow-up, patients were classified into five clinical outcomes (Table 1).

Statistical analysis was done with SPSS v 19.0 software. Epidemiological data is presented as means and standard deviations. We calculated the predictive value to assess the ability of each risk stratification system for predicting the final outcome by performing 2 × 2 contingency tables. The negative predictive value (NPV) was defined as the probability of being free of disease at the end of follow-up in patients who had been classified as low risk by the ATA/ETA systems or had presented an excellent response to initial treatment. We also calculated the positive predictive value (PPV) defined as the probability of persistent disease, either biochemical or structural, and disease specific-mortality, in patients who had been classified as high risk by the ETA system and intermediate or high risk by the ATA system or had presented an acceptable/incomplete response to initial therapy. In order to analyse the ability to predict clinical outcomes, we calculated the area under receiver operating characteristic (ROC) curve for each risk stratification system. We considered a *P* < 0.05 to be statistically significant for all analyses.

## 3. Results and Discussion

### 3.1. Results

All data related to the epidemiological characteristics of the cohort, type of cancer, different risk stratifications, and clinical endpoints are summarized in Table 2.

Most patients initially considered to have low risk of persistent/recurrent disease by static staging systems had no evidence of disease at the end of the study (94% for ETA and 94.9% for ATA system). Despite they were classified as high risk by the ETA system and intermediate risk by the ATA system, they still presented a high likelihood of being free of disease (59.7% for ETA and 62.8% for ATA). However, when patients were initially classified as high risk by the American system, most of them showed negative clinical endpoints (75%). Comparing with the two staging systems, the new risk stratification system showed better predictions of the final outcome. The majority of patients classified as an excellent response showed no evidence of disease at the end of the study (97.7%). On the other hand, those who were classified as an acceptable or incomplete response to initial therapy exhibit higher probabilities of having negative clinical outcomes (68.5% for the acceptable response group and 72.4% for incomplete response group). Furthermore, most patients who presented an acceptable response and had persistent disease showed persistent biochemical disease but no evidence of structural disease. All patients dying of thyroid cancer were correctly classified by the three risk stratification systems (Table 3).

We made 2 × 2 contingency tables to calculate predictive values for each risk stratification system (Table 4). Regarding PPV, ETA and ATA systems presented low PPV, 40.3% and 41%, respectively, whereas RIT system showed acceptable PPV (70.8%). The negative predictive value shown by the RIT system was even higher (NPV 97.7%) than those observed for ETA (93.9%) and ATA systems (94.9%).

**Figure 1:**
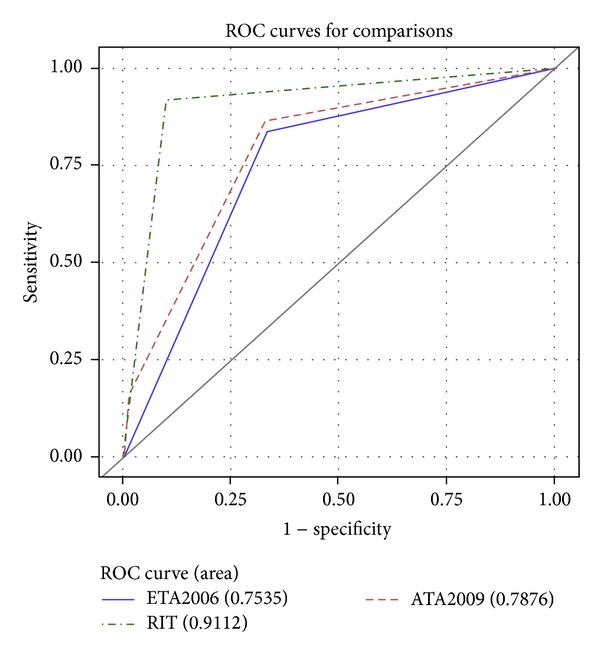
Area under ROC curve to detect recurrence/persistent disease for each risk stratification system.

In order to analyse the ability to predict clinical outcomes, the area under ROC curve was calculated for each risk stratification system and compared between them (Figure 1). The area under ROC curve was 0.7535 for ETA system (95% IC = 0.6816–0.8253), 0.7876 for ATA system (95% IC = 0.7164–0.8587), and 0.9112 for RIT system (95% IC = 0.8584–0.9640), showing statistical significant differences between ongoing staging system and the two static staging systems. There were no statistical significant differences between ATA and ETA systems (Table 5).

### 3.2. Discussion

The major finding in this work suggests that the new ongoing system proposed by Tuttle predicts better the final outcome than the two classical systems and is in agreement with different works published during the last few years. In 2010, Tuttle et al. compared the ability to predict the risk of recurrence/persistence disease by the RIT system and the one proposed by the American association by determining the proportion of variance explained (PVE) and showed 34% of the variance for the ATA system, increasing the PVE to 84% when the new system was used. They also demonstrated the impact of restratification, being more apparent in patients initially classified as intermediate risk, with a risk reduction from 18% to 2%, in those who had presented an excellent response to therapy. Otherwise, patients who presented an incomplete response to therapy experienced an increase in the initial risk (from 3% to 13%) [8]. Castagna et al. proposed in 2011 a new staging system incorporating data at the time of the first control (8–12 months after initial therapy), categorizing patients into delayed risk stratification (DRS) low-risk and DRS high-risk group [9]. They analysed the ability of each stratification system by determining the PPV and NPV and observed very low PPV for both ATA and ETA (39.2% and 38.4%, resp.), while the NPV were rather high (90.6% and 91.3%, resp.). However, DRS system showed significantly better predictive values than the two classical systems with a PPV of 72.8% and a NPV of 96.3%. Finally, in 2013, Jeon et al. incorporated the level of serum TgAb in the dynamic risk stratification proposed by Tuttle and compared with TNM staging and ATA systems and also showed the highest PVE (44.6%) among the three risk stratification systems (8.7% for TNM staging and 12.1% for ATA classification) [10].

In our experience, the incomplete response to initial therapy cohort includes a heterogeneous group of patients, ranging from those with only biochemical persistent disease to those with structural disease, actually exhibiting distinct prognosis. Therefore, in 2011, Vaisman et al. proposed subdiving the incomplete response group into biochemical incomplete response and structural incomplete response, showing that structural incomplete response was associated with higher likelihood of presenting structural evidence of disease and disease specific mortality at the end of follow-up than biochemical incomplete response alone [11]. It would be interesting to apply this modified RIT system to our cohort of 176 patients with differentiated thyroid cancer in future works.

Several authors have demonstrated the usefulness of the American and European systems to predict the risk of recurrence/persistent disease, basically based on initial TNM stage [12]. However, successful ablation, established by negative serum TG and negative ^131^I diagnostic whole body scan, leads to higher probabilities of presenting favourable clinical endpoints regardless of the initial risk, thus supporting the need for new ongoing risk stratification systems [13]. For this reason, they both constitute good systems for tailoring the intensity of initial management and follow-up strategy but lose effectiveness in predicting long-term outcomes. We consider new ongoing risk stratification systems as a complementary tool in order to individualize long-term management and follow-up strategy. For example, in high risk patients with an undetectable stimulated TG and normal findings on neck US and diagnostic ^131^I whole body scan at the 6- to 12-month follow-up, the risk of recurrence is very low. Therefore, subsequent follow-up could be based on suppressed TG and neck US, avoiding additional stimulated TG and diagnostic ^131^I whole body scan, and the dose of L-thyroxine could be safely decreased with the goal of obtaining a thyrotropin level within the lower normal range.

Highly sensitive detection tools used in clinical practice have led to diagnose an important number of patients who have persistent evidence of small-volume disease, mostly detected by stimulated TG measurements without evidence or little structural disease. Although management of these patients remains controversial, additional therapy provides no benefit in overall survival rates. Therefore, we consider that most of these patients can undergo follow-up with observation alone and practice additional therapy in case of evidence of progression.

An important limitation of this study is that all data were evaluated retrospectively. Considering that the majority of recurrences are diagnosed during the first years after initial treatment, a median follow-up period of 7 years seems adequate, although longer studies need to be made in order to validate new dynamic staging systems.

Moreover, this new restratification system can only be applied in patients who underwent total or near-total thyroidectomy and thyroid ablation with I131. Management guidelines do not recommend thyroid ablation in very low risk group of the ETA system and low risk patients of the ATA system with unifocal cancer <1 cm or multifocal cancer when all foci are <1 cm in the absence of other high risk features. For this reason, different staging systems need to be assessed in this population.

Another limitation is the inability to diagnose recurrence/persistent disease by determining thyroglobulinemia in the presence of interfering TgAb. As we mentioned previously, Jeon et al. incorporated TgAb levels in the dynamic risk stratification system, considering a value exceeding 60 IU/mL to be positive for interfering in TG assessment [12]. One criterion considered as an excellent responder was to present a negative TgAb determination. However, in our opinion, lower TgAb concentrations are able to interfere in TG assessment by immunometric methods and, although there is a drastic decrease during the first six months after surgery and it could be useful in order to detect recurrence disease, they could take longer to become undetectable.

Finally, limited number of patients classified as high risk by the ATA system and acceptable/incomplete response by the RIT system induce less consistent predictions of future outcomes in these groups; therefore, it would be interesting to enlarge the number of patients in future works.

## 4. Conclusion

In conclusion, in order to offer appropriate management to patients, ongoing reassessment of the risk of recurrence/persistent disease during follow-up is required. Thereby, our findings provide evidence that response to initial therapy is a useful risk stratification system to adapt and modify the intensity of follow-up, avoiding unnecessary diagnostic tests in those patients who have presented an excellent response, and, on the other hand, being more aggressive in those patients who have presented an incomplete response, regardless of the initial risk.

## Figures and Tables

**Table 1:** Clinical outcomes at the end of follow-up period.

(1) No evidence of disease after initial therapy (i) Undetectable suppressed and stimulated TG (ii) No evidence of structural disease by neck US and diagnostic ^131^I whole body scan (iii) No need for additional therapy	

(2) No evidence of disease after additional therapy (i) Undetectable suppressed and stimulated TG (ii) No evidence of structural disease by neck US, diagnostic ^131^I whole body scan, and other cross-sectional imaging if performed (CT scan, MRI, FDG-PET scan, bone scintigraphy) (iii) Need for additional therapy (surgery, second dose of ablative ^131^I, and chemotherapy)	

(3) Persistent biochemical disease (i) Suppressed TG ≥ 1 ng/mL or stimulated TG ≥ 2 ng/mL (ii) No evidence of structural disease by neck US, diagnostic ^131^I whole body scan, and other cross-sectional imaging if performed (CT scan, MRI, FDG-PET scan, bone scintigraphy)	

(4) Persistent structural disease Any evidence of disease on neck US, diagnostic ^131^I whole body scan, and other cross-sectional imaging (CT scan, MRI, FDG-PET scan, bone scintigraphy) or biopsy proven disease	

(5) Disease specific mortality	

CT: computerized tomography; MRI: magnetic resonance imaging; FDG-PET: fluorodeoxyglucose positron emission tomography.

**Table 2:** Epidemiological characteristics of the cohort.

Characteristics	Mean (SD)
Age	43.4 (14.1)

Characteristics	% (*n*)

Sex	
Male	26.7 (47)
Female	73.3 (129)
Histology	
Papillary classic subtype	51.7 (91)
Papillary follicular subtype	26.1 (46)
Follicular	12.5 (22)
Poorly differentiated/insular	2.8 (5)
Hurthle cell	1.1 (2)
Mixed histology	4 (7)
Papillary oncocytic subtype	1.7 (3)
ETA risk stratification	
Low	56.3 (99)
High	43.7 (77)
ATA risk stratification	
Low	55.7 (98)
Intermediate	39.8 (70)
High	4.5 (8)
RIT risk stratification	
Excellent	72.7 (128)
Acceptable	10.8 (19)
Incomplete	16.5 (29)
Evidence of disease at final follow-up	
No evidence of disease after initial therapy	72.2 (127)
No evidence of disease after additional therapy	6.8 (12)
Persistent biochemical disease	14.2 (25)
Persistent structural disease	5.7 (10)
Disease specific mortality	1.1 (2)

*n* = 176; SD: standard deviation.

**Table 3:** Clinical endpoints for each risk stratification system.

	ETA		ATA			RIT		
	Low (*n* = 99)	High (*n* = 77)	Low (*n* = 98)	Intermediate (*n* = 70)	High (*n* = 8)	Excellent (*n* = 128)	Acceptable (*n* = 19)	Incomplete (*n* = 29)
No evidence of disease after initial therapy	87.9% (87)	51.9% (40)	91.8% (90)	51.4% (36)	12.5% (1)	97.7% (125)	10.5% (2)	0% (0)

No evidence of disease after additional therapy	6.1% (6)	7.8% (6)	3.1% (3)	11.4% (8)	12.5% (1)	0% (0)	21.1% (4)	27.6% (8)

Persistent biochemical disease	5.1% (5)	26% (20)	4.1% (4)	25.7% (18)	37.5% (3)	2.3% (3)	63.2% (12)	34.5% (10)

Persistent structural disease	1% (1)	11.7% (9)	1% (1)	11.4% (8)	12.5% (1)	0% (0)	5.3% (1)	31% (9)

Disease specific mortality	0% (0)	2.6% (2)	0% (0)	0% (0)	25% (2)	0% (0)	0% (0)	6.9% (2)

**Table 4:** 2 × 2 contingency tables for each risk stratification system.

	ETA		ATA		RIT	
	Low (*n* = 99)	High (*n* = 77)	Low (*n* = 98)	Intermediate/high (*n* = 78)	Excellent (*n* = 128)	Acceptable/incomplete (*n* = 48)
No evidence of disease	93.9% (93)	59.7% (46)	94.9% (93)	59% (46)	97.7% (125)	29.2% (14)
Persistent disease	6% (6)	40.3% (31)	5.1% (5)	41% (32)	2.3% (3)	70.8% (34)

**Table 5:** Comparison of the area under ROC curves between the ETA, ATA, and RIT systems.

ROC contrast rows estimation and testing results						
Contrast	Estimate	Standard error	95% Wald confidence limits		Chi-square	Pr > Chi-sq.
ATA2009-ETA2006	0.0341	0.0215	−0.00807	0.0763	2.5130	0.1129
RIT-ETA2006	0.1578	0.0423	0.0749	0.2407	13.9064	0.0002
RIT-ATA2009	0.1237	0.0400	0.0453	0.2021	9.5551	0.0020
